# Foliar Treatments of 2,4-Dichlorophenoxyacetic Acid for Control of Common Scab in Potato Have Beneficial Effects on Powdery Scab Control

**DOI:** 10.1155/2014/947167

**Published:** 2014-05-29

**Authors:** Hannah Katherine Thompson, Robert Stephen Tegg, Ross Corkrey, Calum Rae Wilson

**Affiliations:** Tasmanian Institute of Agriculture, New Town Research Laboratories, University of Tasmania, 13 St. Johns' Avenue, TAS 7008, Australia

## Abstract

Prior studies have shown that applications of the synthetic auxin 2,4-dichlorophenoxyacetic acid (2,4-D) to the foliage of potato plants can reduce common scab. Here field and glasshouse trials suggest that 2,4-D foliar treatments may also reduce the biologically distinct tuber disease, powdery scab. Significant correlations between suppression of common and powdery scab from the field trials suggested an interaction between the two diseases or possible additional broad spectrum mechanisms of enhanced defence against pathogen invasion provided by 2,4-D treatment.

## 1. Introduction

Common scab and powdery scab are two of the most economically important diseases of potato [1]. They exhibit similar tuber symptoms but are pathologically distinct; common scab is caused by infection of developing tubers with pathogenic* Streptomyces *spp. (an Actinomycete) and powdery scab by* Spongospora subterranea* f. sp.* subterranea* (a protozoan) [2, 3]. Management of either disease is problematic with no single reliable control treatment available. In prior studies foliar application of certain substituted phenoxy, benzoic, and picolinic acids to growing potato plants was shown to suppress common scab disease [4–6]. Of the materials tested, one of the most effective was 2,4-dichlorophenoxyacetic acid (2,4-D) [4, 7, 8] more commonly used at higher rates as a herbicide. Further studies showed that 2,4-D treatments were most effective against common scab when applied shortly after crop emergence [9] and that disease suppression was associated with diminished sensitivity of tubers from treated plants to thaxtomin A [7–9], a toxin produced by pathogenic* Streptomyces* spp. shown to be essential for disease expression [10, 11]. This suggested a pathogen specific control mechanism. Contrary to expectations, we observed tubers harvested from 2,4-D treated plants grown under field conditions which appeared to show diminished powdery scab as well as common scab. In this paper we document field and glasshouse trials confirming the effect of 2,4-D treatment on reducing powdery scab severity on potato tubers and suggesting an association between powdery scab and common scab suppression.

## 2. Materials and Methods

### 2.1. Planting Material

Potato tubers of the varieties Russet Burbank (moderately resistant to both powdery scab and common scab) and Desiree (highly or moderately susceptible to powdery scab and common scab respectively) were used. For the field trials, visibly clean seed tubers were either cut into 50 g seed pieces (field trial number 1) or planted whole (field trial number 2). For the glasshouse trial disease-free minitubers produced from tissue-cultured plants were used.

### 2.2. Preparation of 2,4-D Treatments

Crystalline 2,4-D (Sigma Aldrich, St. Louis, MO, USA) was used for the pot trial. Amicide 625 (Nufarm Pty Ltd., Melbourne, Australia) with 625 g/L 2,4-D present as low volatile dimethylamine and diethanolamine salts was used in both field trials to minimise the chance of damage to nearby horticultural crops. Treatment solutions were prepared in water to 100 mg/L and 400 mg/L 2,4-D with a water control. Tween-80 (0.5 g/L) was added as a surfactant to the treatment and control solutions.

### 2.3. Pot Trial

The pot trial was established in December 2012. Pots (20 cm diameter) were filled with pasteurized potting mix (one part course sand, one part peat, and eight parts composted pine bark, pH 6.0). Sp.* subterranea *inoculum was obtained from heavily diseased potato tubers harvested from North West Tasmania, Australia. Peel containing infected skin and underlying tissues up to 1 cm depth from ten tubers was homogenized in a small volume of sterile water using a hand held blender and made up to 2 L total volume with sterile water. A standard aliquot (100 ml) of the constantly agitated inoculum solution was then added to the surface of each pot at time of planting, with the inoculum thoroughly watered in. Mean pathogen content within the trial was estimated by quantitative PCR analysis of a total soil sample of 50 g collected and pooled from three randomly selected pots 15 days after plant emergence (DAE) [13]. The trial had two 2,4-D treatments, 100 mg/L and 400 mg/L, and a water control with plots arranged in a randomised block design with three plants (pots) per cultivar per treatment. Pots were placed outdoors and subject to natural variations in temperature and rainfall with supplemental watering to maintain high soil moisture levels. Treatments were applied to the foliage 20 DAE until runoff. Tubers were harvested following plant senescence 131 DAE and stored at 4°C for 1-2 weeks before disease assessment.

### 2.4. Field Trials

Field trial number 1 was planted in October 2008 at Bishopsbourne, NW Tasmania, on a brown clay soil that had been sown with potatoes in the previous season. The trial was arranged in a randomised split plot design, each plot consisting of two subplots of five plants, one of each variety, with four replicates. Crop emergence (when >80% of plants had emerged) was 23 days after planting. Three 2,4-D spray treatments were applied with a backpack rig to the foliage until run off to each cultivar as single, double, or triple treatments of 100 mg/L 2,4-D with the first application at 10 DAE with subsequent applications at 20 and 30 DAE. Water controls were applied three times (10, 20, and 30 DAE). Plots were irrigated and hand weeded as required. As plants began to senesce the plots were sprayed with a desiccant (Reglone, Syngenta Crop Protection, Cambridge, UK) as per industry standards, with harvest on 2nd April. All the tubers of each variety were collected and combined from the five plants within each plot. Tubers were stored for 1-2 weeks at 4°C prior to being washed and assessed for disease.

Field trial number 2 was planted in October 2010 at Waterhouse, NE Tasmania, on a predominately sandy soil that had been sown with potato crops in the previous two seasons. In this trial only Russet Burbank was used. The trial had two 2,4-D treatments, 100 mg/L and 400 mg/L, and a water control with plots arranged in a randomised block design, each plot consisting of five plants with four replicates. Crop emergence was 24 days after planting. Treatments were applied to foliage at 20 DAE until runoff. The trial site was managed as before, with harvest at 135 DAE. Tubers from each plot were stored for 1-2 weeks at 4°C, washed, and assessed for disease.

### 2.5. Disease Assessment

Tubers >2 g were assessed for disease. Distinctions between lesions of the common scab and powdery scab were generally obvious based on assessor experience with any indeterminate lesions typed by microscopic examination and observation for sporosori associated with* S. subterranea* infections. Severity assessment for both diseases used a tuber surface cover score (0: no visible disease on tuber surface, 0.5: <1%, 1: 1–<5%, 2: 5–<10%, 3: 10–<30%, 4: 30–<50%, 5: 50–<70%, and 6: 70–100% tuber surface affected) with a percentage tuber coverage estimated from the mid values of these score ranges [12].

### 2.6. Data Analysis

Data were analysed using Proc GLM in SAS version 9.3 (SAS Institute Inc., Cary, NC, USA). Multivariate analysis of variance (MANOVA) was used to determine significant effects from and interactions between treatment factors. Significance was determined using the *F* test approximations to Wilk's lambda. The MANOVA also provided an estimate of the partial correlation matrix for the outcome variables with a statistical test that the correlations were zero after adjustment for treatment and block effects. Univariate analyses of variance (ANOVA) for the outcomes were also calculated. Probabilities less than 0.05 were considered significant for the MANOVA results and at the 0.025 level for the ANOVA results to allow for multiplicity of tests. Within the ANOVA results, significant differences between treatments were determined by pairwise comparisons using the Tukey adjusted pairwise comparison of least square means.

## 3. Results

In the glasshouse trial and both field trials common scab and powdery scab were present in harvested tubers. The glasshouse trial had less overall disease compared to the field trials. Pathogen content within the pot trial was estimated at 127 (low risk of disease) and 31,352 pg DNA (moderate to high risk of disease) per gram of soil for* S. scabies* and* S. subterranea, *respectively. No similar pathogen data were obtained from field trial sites. In the glasshouse trial the partial correlation between powdery scab and common scab diseases was not significant (*r* = 0.28, *P* = 0.22, df = 20). MANOVA showed a significant effect for variety (*F*(2,19) = 28.8, *P* < 0.0001) with Desiree having greater combined disease than Russet Burbank and for (2,4-D treatment) rate (*F*(4,38) = 5.46, *P* = 0.0014) with the water control having greater disease than either 100 or 400 mg/L 2,4-D treatment which were not different from each other. There was no significant interaction between variety and rate (*F*(3,38) = 2.27, *P* = 0.08).

Examination of individual disease data by ANOVA showed for powdery scab that there was a significant effect for rate (*F*(2,20) = 12.58, *P* = 0.0003) but not for variety (*F*(1,20) = 2.71, *P* = 0.115) or interaction between rate and variety (*F*(2,20) = 0.92, *P* = 0.416). For common scab, variety (*F*(1,20) = 60.31, *P* < 0.0001), rate (*F*(2,20) = 5.64, *P* = 0.012), and the interaction between variety and rate (*F*(2,20) = 4.48, *P* = 0.025) were all significant (Table 1).

In field trial number 1 the partial correlation between powdery scab and common scab diseases was nonsignificant (*r* = 0.43, *P* = 0.14, df = 12). Similar to the glasshouse trial, MANOVA showed an overall significant effect for variety (*F*(2,11) = 28.2, *P* < 0.0001) with Desiree again having greater combined disease than Russet Burbank and for 2,4-D rate (*F*(6,16) = 7.55, *P* < 0.0001) with the control again having greater disease than either 100 or 400 mg/L 2,4-D rate which were not different from each other (*F*(6,22) = 1.85, *P* = 0.136). Once again there was no significant interaction between variety and rate. Examination of individual disease data by ANOVA showed for powdery scab that there were significant differences between rates (*F*(3) = 17.31, *P* = 0.0004) with all 2,4-D treatments producing less disease than the untreated control. There were no significant differences found between varieties (*F*(1,12) = 0.28, *P* = 0.605) nor was the interaction between rate and variety significant (*F*(3,12) = 0.52, *P* = 0.678). For common scab significant differences for variety (*F*(1,12) = 46.8, *P* < 0.0001) and rate (*F*(3,12) = 38.63, *P* < 0.0001) were found but not for the interaction between variety and rate (*F*(3,12) = 3.34, *P* = 0.056; Table 2).

In field trial number 2 there was a significant correlation between powdery scab and common scab shown (*r* = 0.86, *P* = 0.013, df = 6). Examination of the combined dataset by MANOVA showed no significant effect for treatment (*F*(4,10) = 3.05, *P* = 0.07). Analysis of individual disease data by ANOVA showed for powdery scab that there were no significant differences between rates (*F*(2,6) = 3.93, *P* = 0.081) although a strong trend for greater disease in tubers from the untreated control than from 2,4-D treated plants was suggested (Table 3). For common scab, 2,4-D rate did significantly differ (*F*(2,6) = 7.58, *P* = 0.023) with the control having greater disease than either 2,4-D rate which did not differ from one another (Table 3).

## 4. Discussion

Prior studies have demonstrated that foliar application of low concentrations of 2,4-D (a synthetic auxin) to potato plants prior to tuberization results in reduced incidence and severity of common scab and that this suppression of common scab was associated with a diminished sensitivity of tubers from treated plants to the toxin thaxtomin A produced by pathogenic* Streptomyces* species [4, 7–9]. Furthermore a relationship between thaxtomin A sensitivity in plant tissues and auxin cellular efflux transport processes has been demonstrated [14]. This suggested that the mechanism of disease suppression would be specific to common scab. It was therefore surprising that we have found a significant effect of 2,4-D treatment on powdery scab severity, a disease biologically distinct to common scab [3]. In all trials the pattern of powdery scab suppression appeared similar to that of common scab. In field trial number 1 where incidence of both diseases was greatest, a significant correlation in severity of two diseases was shown. This suggests two possibilities: firstly that the two diseases interact, with infection of one promoting infection of the other perhaps by providing new infection sites; therefore conceivable suppression of common scab by 2,4-D treatment effect on thaxtomin A could lead indirectly to suppression of powdery scab or secondly that the 2,4-D treatments have an inhibitory effect on powdery scab in addition to common scab unrelated to its effect on thaxtomin A. Whilst the association between 2,4-D treatment and thaxtomin A sensitivity appears strong, it is of course possible that 2,4-D treatments may also activate additional defence responses in the plant. Systemic acquired resistance (SAR) responses providing protection against pathogen attack are well known following various chemical treatments of plants. For example, prohexadione-calcium treatment provides a SAR response in pears and apples against fire blight, fosetyl-Al against late blight of potato, and benzothiadiazole and acetylsalicylic acid against early blight, powdery mildew, and* Fusarium* dry rot of potato with treatments generally only effective when the treatments are applied earlier in the growing season or prior to symptom expression [15–18]. Also, treatment of potato plants with the natural auxin IAA has been shown to decrease the severity of late blight [19]. This suggests that 2,4-D treatments may indeed be directly inhibitory to other potato pathogens. Further studies examining 2,4-D influence on powdery scab in absence of the common scab may be required to clarify the associations between these diseases and 2,4-D treatment.

## Figures and Tables

**Table 1:** Glasshouse trial: effect of foliar applications of 100 or 400 mg/L 2,4-D on plants of potato varieties Russet Burbank (RB) and Desiree (DE) on powdery scab and common scab severity.

2,4-D rate (mg/L)	Least square mean of disease cover score*				
	Powdery scab			Common scab	
	RB	DE	Mean (rate)	RB	DE
0	0.50	0.83	**0.66** ^a^	0.16^bc^	2.30^a^
100	0.00	0.25	**0.13** ^b^	0.13^bc^	1.10^b^
400	0.03	0.00	**0.01** ^b^	0.05^c^	1.10^b^

Rate	(*F*(2, 20) = 12.58, *P* = 0.0003)			(*F*(2, 20) = 5.64, *P* = 0.012)	
Variety	(*F*(1, 20) = 2.71, *P* = 0.115)			(*F*(1, 20) = 60.31, *P* < 0.0001)	
Rate × variety	(*F*(2, 20) = 0.92, *P* = 0.416)			(*F*(2, 20) = 4.48, *P* = 0.025)	

*Disease cover score (0–6 scale [12]) assesses surface area of tubers covered with lesions. Percentage cover is the estimated percentage of tuber surface coverage derived from the disease cover score.

Treatment least square means with the same letter within individual disease columns are not significantly different at *P* = 0.025 using the Tukey adjusted pairwise test.

**Table 2:** Field trial number 1: effect of single or multiple foliar applications of 100 mg/L 2,4-D on plants of potato varieties Russet Burbank (RB) and Desiree (DE) on powdery scab and common scab severity.

2,4-D rate (mg/L)	Application dates* (DAE)	Least square mean of disease cover score^†^					
		Powdery scab			Common scab		
		RB	DE	Mean (rate)	RB	DE	Mean (rate)
0	10, 20, 30	3.74	3.73	**3.73** ^a^	3.20	3.35	**3.28** ^a^
100	10	2.79	2.68	**2.74** ^b^	1.57	2.28	**1.92** ^b^
200 (2 × 100)	10, 20	2.70	2.57	**2.64** ^b^	1.48	2.24	**1.81** ^b^
300 (3 × 100)	10, 20, 30	2.63	2.73	**2.68** ^b^	1.35	2.25	**1.80** ^b^

Mean (variety)					**1.90** ^b^	**2.50** ^a^	

Rate		(*F*(3, 12) = 17.31, *P* = 0.0004)			(*F*(3, 12) = 38.63, *P* < 0.0001)		
Variety		(*F*(1, 12) = 0.28, *P* = 0.605)			(*F*(1, 12) = 46.8, *P* < 0.0001)		
Rate × variety		(*F*(3, 12) = 0.52, *P* = 0.678)			(*F*(3, 12) = 3.34, *P* = 0.056)		

*Date (DAE) is the number of days after the average crop emergence.

^†^Disease cover score (0–6 scale [12]) assesses surface area of tubers covered with lesions. Percentage cover is the estimated percentage of tuber surface coverage derived from the disease cover score.

Treatment least square means with the same letter within individual disease columns are not significantly different at *P* = 0.025 using the Tukey adjusted pairwise test.

**Table 3:** Field trial number 2: effect of foliar applications of 100 or 400 mg/L 2,4-D on the foliage of Russet Burbank plants on powdery scab and common scab severity.

2,4-D rate (mg/L)	Least square mean of disease cover score^a^	
	Powdery scab	Common scab
0	1.50	1.18^a^
100	0.87	0.47^b^
400	0.98	0.40^b^

Rate	(*F*(2,6) = 3.93, *P* = 0.081)	(*F*(2,6) = 7.58, *P* = 0.023)

^a^Disease cover score (0–6 scale [12]) assesses surface area of tubers covered with lesions. Percentage cover is the estimated percentage of tuber surface coverage derived from the disease cover score.

Treatment least square means with the same letter within individual disease columns are not significantly different at *P* = 0.025 using the Tukey adjusted pairwise test.
